# The Treatment of Cholera and Infantum

**Published:** 1885-08-20

**Authors:** W. Byford Ryan

**Affiliations:** Indiana


### THE TREATMENT OF CHOLERA INFANTUM.

BY W. BYFORD RYAN, M.D., INDIANA.

Read before the Indiana State Medical Society, May 12, 1885.

If there be any one theme, among the hundreds that perplex us, which stands preeminent in importance, that theme is the treatment of cholera infantum.

Yearly, this perilous disorder almost decimates our infantile population, hundreds of homes contributing of their priceless jewels to heap high the ghastly sacrifice to our ignorance and helplessness, while the mortality of every clime suited to its development bears gloomy witness to the inefficiency of our means for its cure.

Of the 1,700 children, under five years of age, who died of miasmatic diseases, cholera infantum claimed 30 per cent. in this State in the year 1883, and in the same period caused 11 per cent. of the total deaths from disease among children under five years of age.

In the city of St. Louis, last year, 9.2 per cent. of the total mortality from disease was due to cholera infantum. Of deaths under five years of age, from all diseases, 23.32 per cent., and from zymotic diseases 40 per cent. were from this cause.

Trousseau, the prince of clinicians, after giving what he regarded as the very best treatment, says: "You must, however, remember that the cases in which recovery takes place are few in number, death being the usual termination of infantile cholera." Dr. Francis Delafield, of New York, declares that treatment of any kind is very unsatisfactory, and insists that the child be given a change of climate, if possible; the earlier the better; and this though the prostration be so great that the patient is apparently moribund.

In great cities, where people are overcrowded, where heaps of festering filth are constantly accumulating, and where a close, hot and vitiated atmosphere enervates both old and young, the mortality from this disease is simply terrible. Nor are its ravages confined to the poor, the filthy, and the ill-fed denizens of squalid tenement houses; but the children of luxury, in palatial homes, on fashionable avenues, and the rollicking, roseate inhabitants of salubrious farming districts, yield reluctant tribute to this destroyer of the blooming promise of the harvest of our hopes.

It is useless, however, to dwell on the fatality of this affection and the inadequacy of any generally known method of treatment. All acknowledge these, from the greatest to the least in the profession.

It is not my purpose to discuss the many different theories of the origin of cholera infantum, but, rather, to call attention to what is apparent to all observers, and, if possible, to draw from my premises a few plain, common sense conclusions.

You will pardon a brief iteration of the physical condition of a cholera infantum patient, for, though too familiar to you, it will assist in the development of the theory I desire to present for your consideration.

There is no prodromic period, unless we so regard a laxity of the bowels, which is not usually alarming to the family. The invasion is abrupt. The physiognomy of the child quickly undergoes startling, alarming change. In one instance, in my own knowledge, the change was so rapid and complete that last summer a father failed to recognize, and could scarcely be convinced that the little sufferer was the babe he left but slightly unwell twelve hours before. The eyes are sunken, and a bluish line is clearly seen beneath the lower lids. The child cries out as if it were being suffocated; pitch of voice is sharper and higher than usual, resembling the hydrocephalic cry. The skin is cold and almost destitute of tonicity; on being pinched up the fold returns slowly to the normal position. Vomiting is an urgent and distressing symptom. Almost everything swallowed is ejected within a few moments. In addition to the ingesta, which come up unchanged, the vomited matter is serous fluid and bile. The dejecta, which in the beginning were lienteric, change to a greenish serum, in which floats shreds and flakes of a green substance, generally adhering to the diapers; or the discharge may be of a yellow color, resembling the yolk of egg; but they are always absolutely serous, and very frequent. As the disease progresses, the child falls into a stupor, and lies with the eyes partly closed; there is insatiable thirst throughout, all liquids being swallowed with avidity, regardless of their taste. The abdomen is flaccid and sunken; the skin is dry and of a leaden color. In a word, the entire peripheral circulation, especially that of the capillary system, is almost nil.

The causes which lead to this deplorable state are, in my opinion:

1. The enervating influence of excessive heat, producing, as in Asiatic cholera, spasm of the peripheral arterioles.

2. Hyperemia of the gastro-intestinal apparatus, produced (*a*) by chilly nights following excessively warm days, and (*b*) by the influx of blood from the emptying of the surface capillaries.

3. The vulnerability of the gastro-intestinal viscera in the young generally, and especially in those whose digestive organs are enfeebled by premature weaning or by improper food.

Spasm of the arterioles, or, what amounts to the same, paralysis of the trophic nerves, produce peripheral anemia. The congestive influence of chilly nights, added to the emptying of superficial vessels, favors engorgement of the internal vascular system. The atonic condition of the digestive organs, made more vulnerable by premature weaning or improper food, also invites the fugitive blood. Atonic vessels long distended permit the rapid endosmosis of the serum of the blood. Hence, vomiting, diarrhœa, serous dejecta, anemia, excess of fibrin and solids in the blood, and the coagulability of the blood itself, *thrombi* and *emboli*, the plugging of cerebral vessels; hence, death—if, indeed, death do not claim his victim previous to the formation and lodgment of a clot.

If this view of the causes and pathology of cholera infantum be correct, the rational treatment must necessarily be in direct antagonism to the dictum of Hahnemann, and in full accord with its antipode, *contraira contrariis*, which is:

(*a*) To restore the blood supply to the surface, thereby relieving measurably the visceral engorgement.

(*b*) To establish and maintain capillary action of the entire economy, thus arresting extravasation of serum with all its attendant evils.

(*c*) To give tone to the muscular and mucous coats of the bowel.

(*d*) To supply proper nutriment.

These are the indications. Can they be satisfactorily met?

I answer, unequivocally, they can; and, since I have demonstrated practically what I had builded in theory, and subjected to the crucial test by brother practitioners, I have felt impelled to shout "Eureka!" in every assembly of medical men to which I have gone for the past five years.

Nor yet have I any new drug to present, which possesses the powers requisite for the prosecution of a successful warfare against cholera infantum—nor any nostrum or formula even ; but an old weapon (a two-edged sword) with which all are familiar, yet one, so far as I know, not before used as a remedy in this affection. The agent to which I refer is the *atropia* belladonna, which I regard as the remedy *par excellence*, the specific and prime factor about which all other remedial measures should circle as auxiliary; important and useful, but altogether secondary.

Having come to conclusions satisfactory to myself as to the ætiology of infantile cholera, I cast about me for rational means with which to combat existing conditions.

We find peripheral anemia ; belladonna is the most potential means for flushing the superficial capillaries.

We find the vascular system of the intestines and stomach engorged and sieve-like, permitting liquor sanguinis to escape into the lumen of the viscus ; belladonna produces dryness of mucous membranes.

We find extreme irritability of stomach and intestines, giving rise to vomiting and excessive diarrhœa ; belladonna produces partial anæsthesia of these mucus surfaces, and promptly relieves this condition.

We find progressive anemia, produced by endosmosis of serum; belladonna arrests the waste immediately.

Finally, basing the assertion upon actual experiment by myself and those upon whom I have, with the earnestness of positive conviction, pressed the importance of its administration, I can safely say that belladonna will, in every case, arrest both the vomiting and the diarrhœa at once, and that no child sick of this dread summer complaint, who has a fair constitution, need be lost if it have this treatment, combined with, and followed by, such tonic measures and nourishment as will suggest themselves to any intelligent physician.

Minute doses of nux vomica and arsenic I regard almost as essential as tonic treatment. I refrain from suggesting formulæ, but can not close my remarks without protesting against the use of mercurials in a disorder where there is no lack of bile secretions, and where the blood is being rapidly broken down without the help of agents which produce that effect.

I should be glad if such of my hearers as may be called on to treat either one of the several choleras would give belladonna a full trial, and report results.—*Ind. Med. Jour.*

